# Invasive Trends of *Spartina alterniflora* in the Southeastern Coast of China and Potential Distributional Impacts on Mangrove Forests

**DOI:** 10.3390/plants12101923

**Published:** 2023-05-09

**Authors:** Jiaying Zheng, Haiyan Wei, Ruidun Chen, Jiamin Liu, Lukun Wang, Wei Gu

**Affiliations:** 1 School of Geography and Tourism, Shaanxi Normal University, Xi’an 710119, China; zhengjiaying@snnu.edu.cn (J.Z.); crd@snnu.edu.cn (R.C.); liujiamin_gis@snnu.edu.cn (J.L.); 192023@snnu.edu.cn (L.W.); 2 National Engineering Laboratory for Resource Development of Endangered Crude Drugs in Northwest China, Shaanxi Normal University, Xi’an 710119, China; 3 College of Life Sciences, Shaanxi Normal University, Xi’an 710119, China

**Keywords:** *Spartina alterniflora*, mangrove forests, species distribution models, ensemble model, invasive risk

## Abstract

Mangrove forests are one of the most productive and seriously threatened ecosystems in the world. The widespread invasion of *Spartina alterniflora* has seriously imperiled the security of mangroves as well as coastal mudflat ecosystems. Based on a model evaluation index, we selected RF, GBM, and GLM as a predictive model for building a high-precision ensemble model. We used the species occurrence records combined with bioclimate, sea–land topography, and marine environmental factors to predict the potentially suitable habitats of mangrove forests and the potentially suitable invasive habitats of *S. alterniflora* in the southeastern coast of China. We then applied the invasion risk index (IRI) to assess the risk that *S. alterniflora* would invade mangrove forests. The results show that the suitable habitats for mangrove forests are mainly distributed along the coastal provinces of Guangdong, Hainan, and the eastern coast of Guangxi. The suitable invasive habitats for *S. alterniflora* are mainly distributed along the coast of Zhejiang, Fujian, and relatively less in the southern provinces. The high-risk areas for *S. alterniflora* invasion of mangrove forests are concentrated in Zhejiang and Fujian. Bioclimate variables are the most important variables affecting the survival and distribution of mangrove forests and *S. alterniflora*. Among them, temperature is the most important environmental variable determining the large-scale distribution of mangrove forests. Meanwhile, *S. alterniflora* is more sensitive to precipitation than temperature. Our results can provide scientific insights and references for mangrove forest conservation and control of *S. alterniflora*.

## 1. Introduction

With the rapid development of China’s economy, especially the growth of trade and transportation, biological invasions are occurring very frequently [1,2]. China has become one of the countries in the world with the most serious damage from biological invasions. At the end of 2018, there were nearly 800 invasive alien species in China; 638 species have been confirmed as having invaded agricultural and forestry ecosystems [3]. *Spartina alterniflora* is a perennial herb that originates from the Atlantic coast and Gulf coasts of North America [4] It has successfully invaded coastal wetland areas worldwide through intentional or unintentional introduction by human beings [5,6,7,8]. China introduced *S. alterniflora* in 1979 to protect against wind, siltation, reclamation, and to improve beach vegetation cover and productivity [9,10]. Since its introduction in China, *S. alterniflora* has become the most important invasive plant in coastal wetlands due to its high adaptability and reproductive capacity [11]. The outbreak scale of *S. alterniflora* in China is much larger than in other countries and regions of the world [12].

Mangrove forests are mainly found in the intertidal zone of the world’s coastal tropics and subtropics, acting as buffer zones between land and sea [13,14]. They are among the most productive ecosystems in the world [15,16], having not only great social, economic, and ecological value, but also stabilizing coastlines to reduce the damaging effects of natural disasters [17]. Mangrove forests also provide food, medicine, fuel, and building materials for people as well as important social and economic services such as forest products for residents [18]. When it comes to maintaining and protecting tropical and subtropical marine biodiversity, mangrove forests play an important role [19,20]. However, mangrove forests are one of the most threatened ecosystems [21], especially in Asia and the Pacific, where 70% of their original habitats have been lost [22]. As an important ecological barrier, the survival and distribution of mangrove forests are influenced by climate and environmental changes [23]. At the same time, human activities, such as urban development, aquaculture, mining, over-exploitation of timber, and invasive alien organisms [24,25,26,27,28], have led to extensive degradation of mangrove forests.

Although *S. alterniflora* has played a role in promoting siltation, land reclamation, and soil improvement, its continuous expansion has also brought about more serious ecological consequences and economic losses. Due to its adaptability and rapid growth, *S. alterniflora* has invaded the native mangrove forests along the southern coast of China and is likely to occupy increasing areas of mangrove forest habitat in the future [27]. Different areas of *S. alterniflora* have rapidly occupied most of the outer edges of mangrove forests and significantly inhibited the regeneration of native mangrove plants [29] while threatening the security of coastal mudflat ecosystems. Current research on mangrove forests has focused on the physiological characteristics of plants, chemical composition extraction, benthic biodiversity distribution, genome identification and evolution, and ecological restoration engineering [30,31,32,33]. Studies on *S. alterniflora* have focused on the dynamic responses of *S. alterniflora* to tidal flat systems, native community diversity, food webs, and trophic structure [34,35]; the biogeochemical cycling processes of *S. alterniflora* in salt marshes [36,37,38]; the effects of climate change on the physiological characteristics, geospatial changes, and expansion rates of *S. alterniflora* [39,40]; and the inter-population interactions of the ecosystems in which *S. alterniflora* is located [41,42]. Previous studies have mainly investigated the mechanisms of mangrove forest invasion by *S. alterniflora* at microscopic scales, including differences in chemical substances in sediments of different plant habitats; physical and chemical responses in ecosystems; intertidal benthic differences at small scales [43,44,45]; and the suitability distribution of a particular species alone [46,47]. The spatial areas of mangrove forest invasion by *S. alterniflora* at a large scale have been less studied.

Climate is an important environmental determinant of species distribution, and climate change has significant impacts on biodiversity, including species distribution and interspecific interactions [48,49]. The IPCC has finalized the first part of the *Sixth Assessment Report*, which states that the climate system is expected to continue warming by mid-century, with climate change bringing many different combinations of changes to different regions [50]. As a result, an increasing number of studies have focused on how climate change affects species distribution, while also considering a variety of other factors, such as topography, soils, and human activities [51,52,53,54,55,56]. However, species located in coastal areas are not only subject to climate change, but also the marine environment. Factors such as sea surface temperature, sea surface salinity, photosynthetically available radiation, and water quality can directly or indirectly affect the distribution of coastal wetland plants [15,57].

Species distribution models (SDMs) are widely used to predict the potential distribution of species [58]. SDMs are used to visualize the association of species occurrence records and environmental variables through functional relationships to form various algorithms and predict the potential distribution of target species [59]. Various SDMs have been developed based on the rapid development of computer software and technological innovation. Commonly used SDMs are maximum entropy models (MaxEnt) [60,61], generalized linear models (GLM) [62], generalized add models (GAM) [63], classification tree analysis (CTA) [64], artificial neural networks (ANN) [65], flexible discriminant analyses (FDA) [66], generalized boosting models (GBM) [67], random forests (RF) [68], surface range envelopes (SRE) [69], multiple adaptive regression splines (MARS) [70,71], support vector machines (SVM) [72], and maximum likelihood (Maxlike) [73]. A single model may lead to variation in suitable habitats for the same species due to multiple factors, resulting in uncertainty in prediction results. The proposed ensemble model (EM) fixes this problem [53]. The advantage of EM is that it reduces or even eliminates the over-fitting caused by some powerful single algorithms in an integrated manner, significantly enhancing modeling accuracy and reducing model-fitting uncertainty [74,75]. Biomod2 is a collection of programs developed in R for building SDMs. It can be used to simulate the potential distribution of species by building a single model or EM to explore the relationship between species spatial distribution and environmental variables and calibrate and evaluate models [76].

In this study, we used 1358 mangrove forest and 1314 *S. alterniflora* occurrence records from the southeastern coast of China to simulate the potential distribution areas using the EM with terrestrial and marine environmental factors. Then, we applied an invasion risk index (IRI) and analyzed the invasive risk of *S. alterniflora* in the mangrove forest distribution area. Our specific objectives are to (1) select multiple single models for simulation and construct the EM by selecting a single model with a high accuracy of evaluation index; (2) identify the most important environmental variables affecting the distribution of mangrove forests and *S. alterniflora*; (3) predict potentially suitable habitats of mangrove forests and potentially suitable invasion habitats of *S. alterniflora*; (4) analyze the risk of invasion by *S. alterniflora* into mangrove forests in the southeastern coast of China. This study is based on the premise hypothesis of SDM construction: Species distribution and the environment are in an equilibrium stage. The locations where species occur represent suitable environmental spaces and those where they do not occur represent unsuitable spaces. The results of this study can provide the scientific reference for mangrove forest conservation and *S. alterniflora* control.

## 2. Results

### 2.1. Model Evaluation

**Figure 1:**
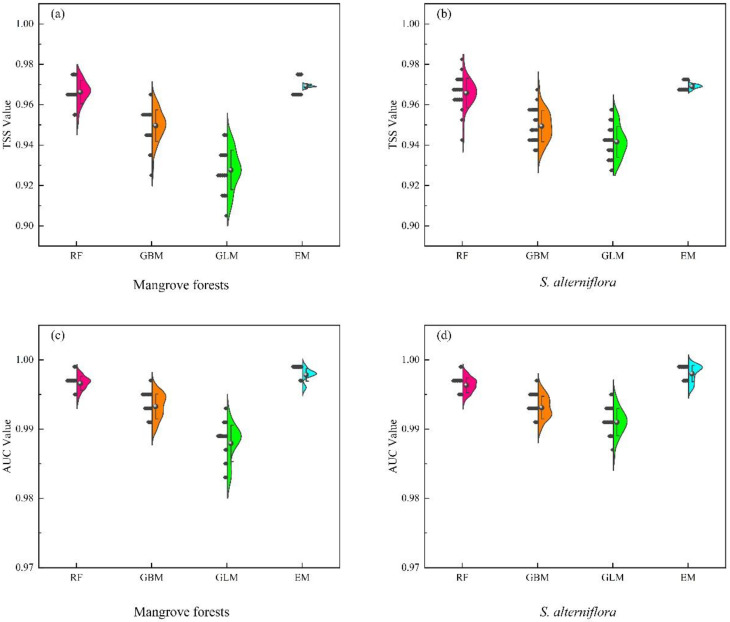
Model evaluation of the single (RF, GBM, GLM) and the ensemble model (EM). The sphere in the graph represents the average value. (**a**) is the TSS value of mangrove forests. (**b**) is the TSS value of *S. alterniflora*. (**c**) is the AUC value of mangrove forests. (**d**) is the AUC value of *S. alterniflora*.

We simulated nine single models and selected RF, GBM, and GLM according to their TSS values for modeling. Their TSS values were all superior to the other six single models. RF, GBM, and GLM were combined to build the ensemble model (EM). Then, we evaluated the model accuracy of RF, GBM, GLM, and EM. The results showed that the ranking of the mean value of TSS of each model was EM > RF > GBM > GLM; the ranking of the mean value of AUC of each model was the same as TSS (Figure 1). The simulation accuracy of the EM is better than that of the single model as can be obtained from the results of the two model metrics, i.e., TSS and AUC. From the simulation results, it can be concluded that the integrated EM with a single model of high accuracy has a higher simulation accuracy.

### 2.2. Analysis of Environmental Variables

We derived the contribution of each variable through EM and analyzed the dominant variables (Table 1). The cumulative contribution rates of bioclimate variables of mangrove forests and *S. alterniflora* were 52.57% and 56.75%; the marine environment variables were 29.44% and 34.18%; and the sea–land topography variables were 17.99% and 9.07%, respectively. Specifically, bioclimate variables played a crucial role in the distribution of mangrove forests and *S. alterniflora*. The role of Ele in sea–land topography variables was relatively large. Among the marine environment variables, only CHL and PAR would play a dominant role in the distribution of mangrove forests and *S. alterniflora*. The contributions of sea surface temperature (SST1, SST2, SST3, SST4, and SST5) and sea surface salinity (SSS) were both low. Among the variables whose cumulative contribution rate reached more than 90%, mangrove forests had 10 key variables, and *S. alterniflora* had 11 key variables (Table 1). Eight variables were common, namely Bio2, Bio12, Bio15, Bio16, Bio19, Ele, CHL, and PAR, but they played different roles in influencing the growth and distribution of mangrove forests and *S. alterniflora* (Figure S2). Among the bioclimate variables, Bio1 played a key role in the prediction of mangrove forests and was the most important temperature variable with a contribution of 25.07% and an optimal threshold of 18.7–25.7 °C. For *S. alterniflora*, the proportion of precipitation reached approximately 3.3 times that of temperature. Bio16 was the highest contributing precipitation variable, with a contribution of 21.27% and an optimal threshold of 438–1226 mm. Among the marine environment variables, CHL was the key variable affecting mangrove forests and *S. alterniflora*: the contribution of CHL was 17.84% and 22.34%, with optimal thresholds of 1.17–13.14 µg/L and 3.04–13.85 µg/L, respectively. The contribution of PAR to mangrove forests and *S. alterniflora* was 3.18% and 5.92%, respectively. The contribution of SST2 to mangrove forests was 5.14%, SST1 to *S. alterniflora* was 2.31%, and the contributions of other marine environment variables were very low. Among the sea–land topography variables, only Ele had a relatively high influence on the distribution of mangrove forests and *S. alterniflora*, with a contribution of 17.86% and 8.29%, respectively. Slop and Aspe had no effect (Table 1).

### 2.3. Simulation Analysis of Potential Distribution for Mangrove Forests and S. alterniflora

**Figure 2:**
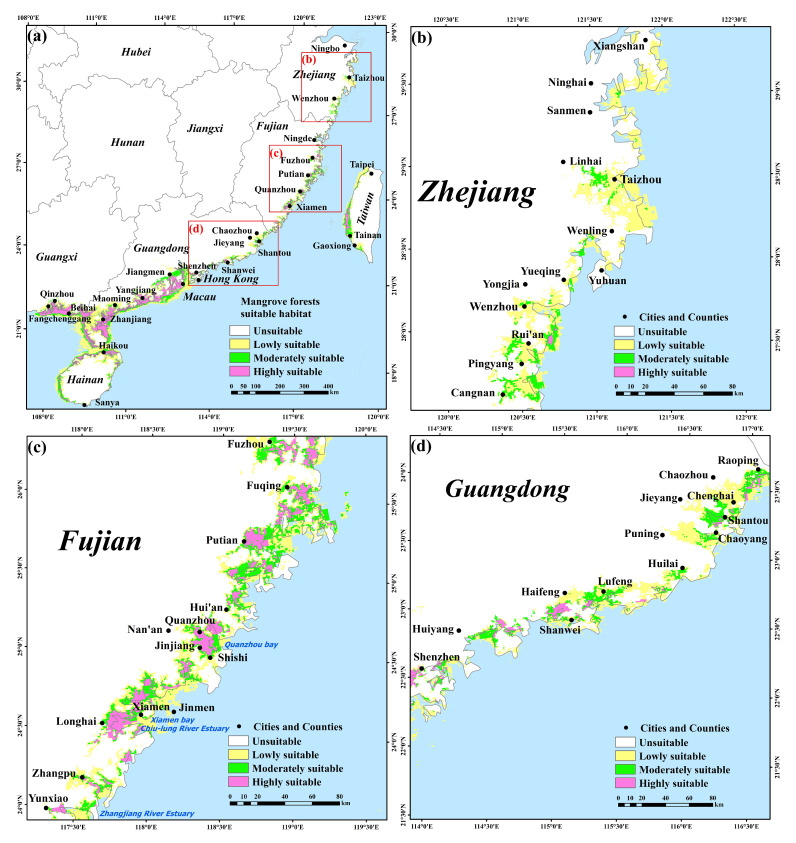
Potential distribution predicted for mangrove forests covering the southeastern coast of China based on the EM. (**b**–**d**) are local enlargements of (**a**).

In this study, the EM prediction results show that the suitable areas of mangrove forests are mainly distributed in eight provinces and regions along the southeastern coast of China, including Guangxi, Guangdong, Hainan, Fujian, Zhejiang, Taiwan, Hong Kong, and Macau (Figure 2a). Highly suitable habitats for mangrove forests are mainly found in harbors or estuaries that are well covered by waves. Specifically, highly suitable mangrove forest habitats in Guangxi are mainly distributed on the southern coast of Beihai city, the east coast of Fangchenggang city, and the coastal harbors and bays of Qinzhou city. In Guangdong province, they are mainly distributed in the coasts of Zhanjiang, Maoming, Yangjiang, and Jiangmen city; other coastal areas, such as Shenzhen city, Shanwei city, and Shantou city, are sporadically distributed (Figure 2d). Most coastal areas in Hainan province are highly adaptable to mangrove forests, including the coast of Haikou city, where mangrove forest nature reserves, such as Dongzhai Port and Qinglan Port, are located. Mangrove forest highly suitable areas are distributed in all coastal sections of Fujian province, such as the Zhangjiang River estuary, Chiu-lung River estuary, Quanzhou Bay, and Xiamen Bay (Figure 2c). Among these, Ningde city is the northernmost boundary of the natural distribution of mangrove forests in China while the rest of the coast has a sporadic distribution. The highly suitable habitats in Taiwan are mainly distributed in the west coast and concentrated in the coastal areas of Taipei freshwater estuaries and the northern area of Tainan City (Figure 2a). Zhejiang is the northernmost distribution area of mangrove forest introduction and cultivation in China, with relatively few highly suitable habitats areas that are sporadically distributed in and around Ximen Island of Yueqing city and the Rui’an coast of Wenzhou city (Figure 2b). The moderate and low suitable habitats of mangrove forests are concentrated around the highly suitable habitats and spread around from the highly suitable areas, with a wide range that extends to Taizhou, Ningbo city in Zhejiang at the northern end.

**Figure 3:**
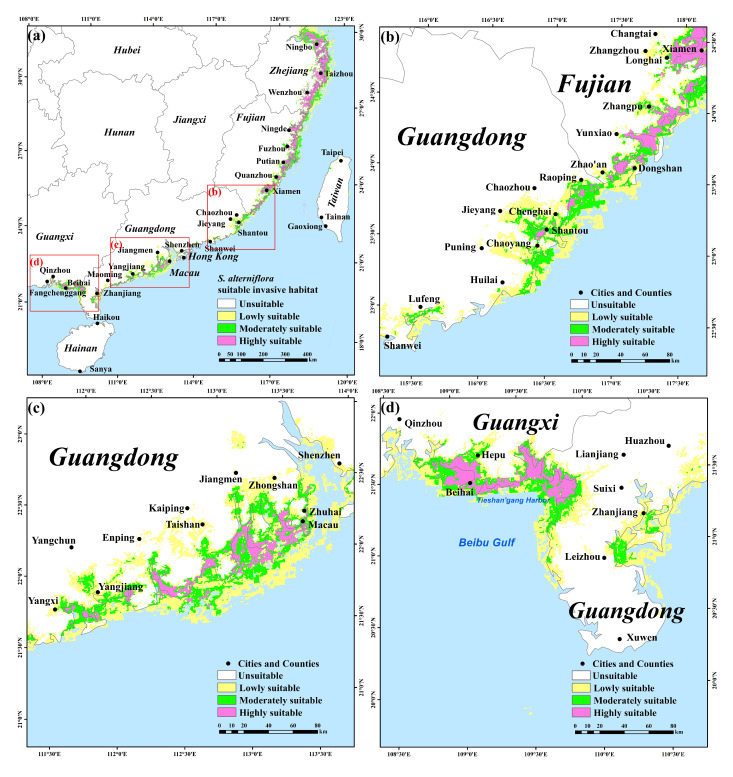
Potential distribution for predicted *S. alterniflora* covering the southeastern coast of China based on the EM. (**b**–**d**) are local enlargements of (**a**).

The results of the EM prediction show that in this study area, the Zhejiang and Fujian coastal areas are the concentrated distribution areas of highly suitable habitats for *S. alterniflora*. Most of the coasts are distributed, the harbor and estuary areas are concentrated, and the width of the distribution strips is wide (Figure 3a). The highly suitable habitats for *S. alterniflora* are scattered in the Fangchenggang, Qinzhou, and Beihai coastal areas of Guangxi Province, and the Zhanjiang, Maoming, Yangjiang, Jiangmen, and Shantou areas of Guangdong Province (Figure 3b–d). In the Zhejiang and Fujian coastal areas, the moderate and low suitable habitats of *S. alterniflora* are mainly at the periphery of the high suitable habitats and extend towards the sea (Figure 3a). In the coastal areas of Zhuhai and Macau, the highly suitable habitats of *S. alterniflora* have not formed a strip, and the moderate and low suitable habitats have a bigger distribution area (Figure 3c). In Guangxi, the highly suitable habitats are mainly in the surrounding areas, and the moderate and low suitable habitats are less distributed (Figure 3d).

### 2.4. Risk Analysis of S. alterniflora Invades Mangrove Forests along the Southeastern Coast of China

**Figure 4:**
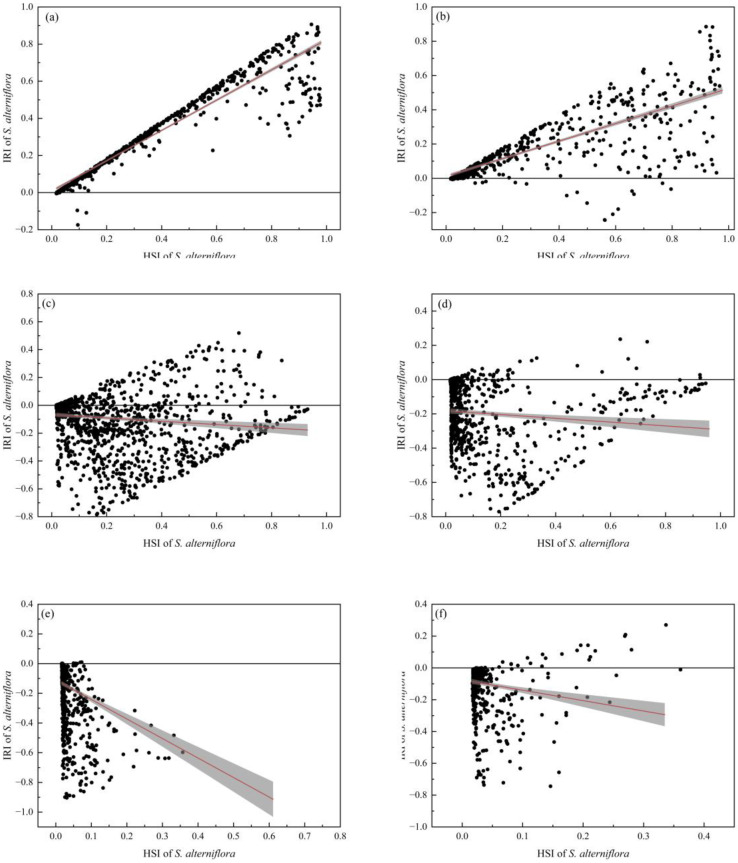
The relationship between invasion suitability and invasion risk of *S. alterniflora* to mangrove forests in the southeastern coast of China. (**a**) Zhejiang, (**b**) Fujian, (**c**) Guangdong, (**d**) Guangxi, (**e**) Hainan, and (**f**) Taiwan. The horizontal axis represents the HSI of *S. alterniflora*, and the vertical axis represents the IRI of *S. alterniflora* to mangrove forests. The red line shows the result of the linear fit; the gray band indicates a 95% confidence band.

**Figure 5:**
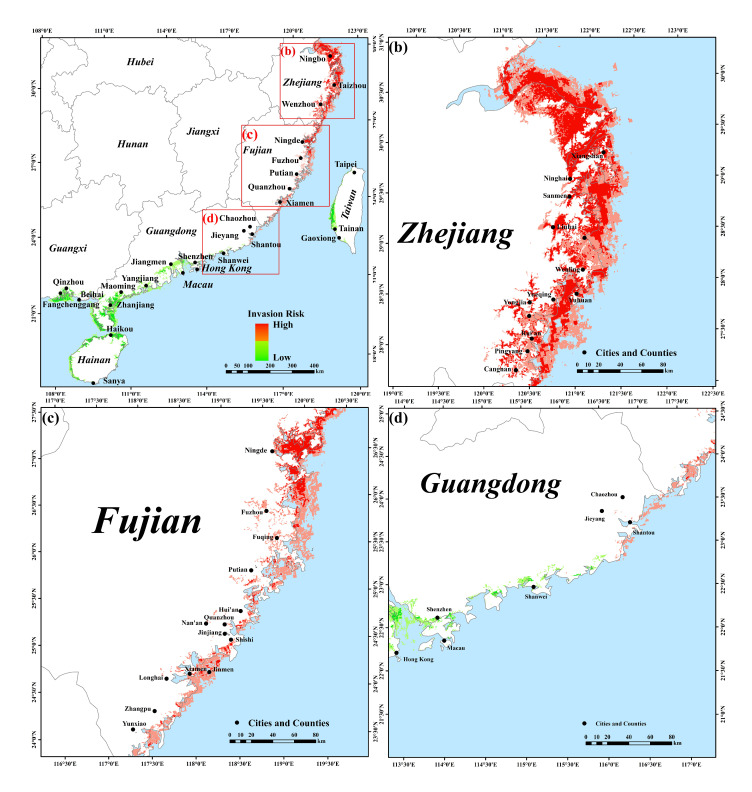
Invasion risk analysis of *S. alterniflora* to mangrove forests in the southeastern coast of China. Red is the area with high invasion risk, and green is the area with low invasion risk. (**b**–**d**) are local enlargements of (**a**).

In the coastal areas of Guangdong, Guangxi, and Hainan, the potential suitability of mangrove forests is higher than that of Fujian and Zhejiang, while the high suitability areas of *S. alterniflora* are mostly distributed in the coastal areas of Fujian and Zhejiang. The potential distributions of mangrove forests and *S. alterniflora* have overlapping ecological niches in Zhejiang, Fujian, Guangdong, and Guangxi provinces (Figure 2a and Figure 3a). In Zhejiang and Fujian, the distribution of *S. alterniflora* is very concentrated and the invasive suitability is high. In the range from low to high potential invasion suitability of *S. alterniflora*, the invasive risk to native mangrove forests is relatively high (Figure 4a,b). In Guangdong and Guangxi, the distribution of highly suitable invasive habitats for *S. alterniflora* is small, and the invasive risk to native mangrove forests is relatively low in areas with high potential invasion suitability for *S. alterniflora* (Figure 4c,d). In Hainan and Taiwan, the distribution of highly suitable invasive habitats of *S. alterniflora* and the invasive risk to native mangrove forests are low (Figure 3a and Figure 4e,f). Specifically, the areas with high risk of invasion of *S. alterniflora* are concentrated in the Zhejiang and Fujian coasts (Figure 5a). The risk in northern Zhejiang is higher than that in southern areas (Figure 5b). Compared with Zhejiang, the invasion risk area along Fujian is narrower (Figure 5c). The risk of invasion of *S. alterniflora* is sporadically distributed in Chaozhou, Shantou, and the Jieyang coast of Guangdong (Figure 5d). Meanwhile, the risk of invasion of *S. alterniflora* in the rest of the region is low.

## 3. Discussion

### 3.1. Applicability of SDMs

SDMs mainly use species distribution data and environmental data to estimate the ecological niches of the species base through specific algorithms. They are then projected onto the landscape to reflect the preference of species for habitats in the form of probabilities [77,78]. In recent years, SDMs have been widely used for the species response to climate change in the context of global change [51]; for potential range prediction of invasive species [52]; for determining the effects of regional climate change on species richness and community stability [79]; for range delineation of protected areas for endangered and rare species; and for determining the impact of human activities on endangered species [80]. In the modeling process for different purposes, species ecological niche characteristics, and modeling database, researchers need to choose different modeling algorithms. In recent years, EM prediction using multiple model information has become a trend in species distribution studies to reduce model uncertainty and increase modeling accuracy [80,81]. Therefore, it is reasonable to use EM in this study.

### 3.2. Ensemble Model Simulation Accuracy

A single model may result in differences in suitable habitat for the same species due to multiple factors. Different algorithms have different architectures and input data assumptions, such as species ecological characteristics, environmental complexity, and data availability, which can result in uncertainty in prediction results [53,82]. Simulations using a single model inevitably produce under-fitting or over-fitting problems, but the EM can reduce the uncertainty of model fitting [52,83]. The EM can combine the predictive strengths of multiple models, reduce the weaknesses of individual models, improve overall predictive accuracy, and effectively address the uncertainty of model extrapolation [84,85,86]. In this study, we selected the TSS > 0.9 models to construct the EM by using a weighted average algorithm to predict the suitable habitats of species. The evaluation metrics of the EM were generally higher than those of the three single models (Figure 1). We can conclude that the model integrated by the good single algorithms can be more reliable and overcome the degree of uncertainty in algorithm selection with higher prediction accuracy and better fitting results.

### 3.3. Selection of Environmental Factors

SDMs is a method for predicting the potential habitats of species by establishing relationships between species distribution points and environmental factors [56]. Therefore, understanding the complexity between species and their environmental factors as well as the selection of appropriate environmental variables are key to building good models [15,86]. In most studies of SDMs, only climatic variables are selected as environmental variables [86], or factors such as climate, topography, and soil are selected as environmental variables [51,82]. Mangrove forests and *S. alterniflora* belong to the intertidal wetland vegetation of coastal mudflats. In this study, two major dual influencing factors, i.e., the terrestrial and marine environments, were considered based on the growth environment of mangrove forests and *S. alterniflora* and previous research results [15,46,57]. Considering the correlation between climate variables, the main climate variables were selected, while the topographic data, as well as sea surface temperature, sea surface salinity, and water quality factors representing the marine ecological environment were selected for predictive simulations. The results of the study show that both terrestrial and marine environmental factors have different degrees of influence on coastal wetland vegetation (Table 2). The response of coastal plants, including mangrove forests and *S. alterniflora*, to factors such as bioclimatic, elevation, and the marine environment, is attributed to the fact that the environmental and material supply required for growth is obtained mainly from both marine and terrestrial sources [15]. Environmental factors, such as temperature, precipitation, salinity, and topography, are the main factors that control the distribution and growth of mangrove forests and *S. alterniflora* in the Chinese coastal zone [87].

### 3.4. Uncertainty in Species Distribution Model Simulations

In the process of simulating the spatial pattern of species, uncertainties in the simulation results are often caused by a variety of factors, such as species distribution data [88], subjectivity and multi-collinearity of environmental variables [89], selection of species distribution models and setting of parameters [90,91], etc. In this study, we chose to use remote sensing data to obtain species point distributions and selected a single model with high model accuracy to construct EM and run it several times to reduce the uncertainty of model prediction. However, the prediction of species distribution still has a large amount of uncertainty, and the distribution of suitable habitats for species changes due to a variety of factors, such as the physical properties of the environment, resource demand, and human activities. Further exploration on how to reduce the uncertainty of species distribution prediction is needed in future studies.

### 3.5. Important Variables Affecting Mangrove Forests and S. alterniflora

Previous studies have shown that temperature is a key factor affecting the distribution of mangrove forests, and that temperature affects the growth and reproduction of mangrove forests [46]. The annual average temperature of the mangrove forest distribution area in China is 19–26 °C, the average temperature of the coldest month is 7.4–21 °C, and the average annual precipitation is 1200–2200 mm [92]. This is consistent with the conclusion reached in this study (Table 1). If the temperature rises by 2 °C, the distribution area of mangrove plants in China will likely expand northward by about 2.5°, and the northern boundary of the introgression can reach Hangzhou Bay from the current location in Yueqing County, Zhejiang Province [93,94]. Currently, with the warming climate, the northern boundary of the introduced mangrove forests has reached Wenzhou and Taizhou, Zhejiang Province [93]. In the present study, the northern boundary of the highly suitable distribution area of mangrove forests predicted by the EM can reach Wenzhou and Taizhou, Zhejiang Province (Figure 2). This is consistent with the results of previous studies [93]. In addition to the effect of temperature, Ele, CHL, and precipitation have an influence on the growth of mangrove forests, and studies have shown that mangrove forests are also more suitable to grow in lowland coastal and water body eutrophic level relatively high areas [15,95,96]. Precipitation can regulate nutrient uptake and thus affect mangrove forest productivity [97], and seasonal precipitation can lead to changes in mangrove forest habitat suitability [98].

*S. alterniflora* is highly suitable and tolerant to climate and environment. Studies have shown that *S. alterniflora* is subject to multiple stresses from precipitation patterns, sea level rise, and nutrient enrichment [99]. Higher nutrient concentrations and increased eutrophication of habitats promote the invasion and expansion of *S. alterniflora* populations [10,100,101]. Precipitation affects the growth of *S. alterniflora* mainly by affecting the salinity of soil pore water, and higher precipitation promotes the growth of *S. alterniflora* [102,103].

### 3.6. Potential Distribution of Mangrove Forests and S. alterniflora

Mangrove forests are mainly located in the warm and humid subtropical and tropical monsoon regions in some harbors or estuaries with good wave cover. Hainan, Guangdong and Guangxi account for about 96% of China’s mangrove forest areas, with small areas in Fujian, Taiwan, Hong Kong, and Macau and no natural distribution of mangrove forests in Zhejiang, which were artificially introduced after the 1950s [24]. From the predicted results, the potentially suitable habitats for mangrove forests are mainly distributed in eight provinces and regions in China, with the northern end reaching Taizhou city, Zhejiang province, and the southern end reaching Sanya city, Hainan province (Figure 2a). This is consistent with previous results [87,92,104,105,106].

According to the survey, the area of *S. alterniflora* in Jiangsu, Zhejiang, Fujian, and Shanghai accounts for 94% of the total distribution area of the national coastal zone; this is the most concentrated distribution of *S. alterniflora* in China [107]. The Zhejiang and Fujian provinces have winding coastlines and easy to form harbors, which provide favorable conditions for the growth of *S. alterniflora* [11]. In this study, the highly suitable invasive areas for *S. alterniflora* are mainly distributed in the coastal areas of Zhejiang and Fujian. Most of the coasts in these two provinces are distributed, with continuous distribution in the harbor and estuary areas. The width of the distribution strip is wide and extends towards the ocean (Figure 3a).

### 3.7. S. alterniflora Invades Mangrove Forests along the Southeastern Coast of China

At present, *S. alterniflora* invasion seriously threatens the mangrove forest resources in Fujian and Guangdong, China and replaces the mangrove forest communities in some areas [11,108]. Previous studies have shown that *S. alterniflora* has a wider range of salinity adaptations, greater flood tolerance, and more rapid reproductive dispersal than native mangrove plants [12,27]. Thus, *S. alterniflora* can spread rapidly in mangrove forest communities and form significant competitive exclusion to mangrove plants, affecting the renewal and growth of mangrove forest seedlings. *S. alterniflora* causes not only degradation of mangrove habitats, but also changes the biodiversity and behavioral patterns of mangrove forests [109]. In this study, we used an invasion risk index (IRI) to assess the invasive risk of *S. alterniflora* in mangrove forest distribution areas along the southeastern coast of China. Zhejiang and Fujian are at high risk of invasion, while other southern provinces are at lower risk of invasion (Figure 5a). This is because Zhejiang and Fujian are the areas with the most invasive *S. alterniflora*. Mangrove forests in the southern provinces of Guangdong and Guangxi are relatively intact and can withstand the invasion of *S. alterniflora* [110]. Through the analysis of the IRI results, mangrove forest protection and restoration using *S. alterniflora* control plans and measures can be set more intuitively. This has significance as a practical reference.

### 3.8. Conservation of Mangrove Forests and Control of S. alterniflora

Mangrove forest wetlands are rich in biodiversity and are one of the most productive ecosystems in the world [15]. Due to its high environmental adaptability and rapid population spread, *S. alterniflora* poses a serious threat to native coastal ecosystems [43]. One of the major causes of the extensive destruction of mangrove forests in China since the 1980s is the invasion of *S. alterniflora* [26]. Therefore, mangrove forest conservation and *S. alterniflora* control are urgent. Based on the results of the current study, we propose the following recommendations for future mangrove forest conservation and *S. alterniflora* control along the southeast coast of China.

#### 3.8.1. Rationalize Mangrove Forest Protection Actions

With an increasing number of people recognizing the unique ecological value of mangrove forests, their restoration and protection have received great attention. In recent years, China has made positive progress in mangrove forest restoration and initially reversed the trend of drastic decrease in mangrove forest area [24,111]. In 2020, China’s National Forestry and Grassland Administration issued a plan called “Special Action Plan for Mangrove Forest Protection and Restoration (2020–2025)”, which required the comprehensive strengthening of mangrove forest protection and restoration work [112]. According to the results of our study, the highly suitable distribution areas of mangrove forests are mainly concentrated in southeastern Guangdong, southern Guangxi, and northern Hainan. Therefore, mangrove forest protection and restoration work should be actively carried out in Guangdong, Guangxi, and Hainan, including the establishment of nature reserves, mangrove forest transplantation, cultivation of new species, and other measures. At present, with the increasing awareness of the social and ecological value of mangrove forests, the introduction of mangrove plants for artificial planting in Zhejiang has also achieved great results, and the northern boundary of the introduction has reached Wenzhou and Taizhou, Zhejiang [92]. At the same time, as the temperature rises, the distribution area of mangrove forest plants will probably expand about 2.5° north, and the northern boundary of the introduced species could reach Hangzhou Bay [92,93]. Mangrove forest priming projects should be planned rationally in the Zhejiang and Fujian areas, using the high suitable distribution area of mangrove forests as reference.

#### 3.8.2. Control *S. alterniflora* According to Local Conditions

The rapid and effective control of *S. alterniflora*, by limiting its proliferation rate and scale and minimizing its ecological damage and impact, have become important issues to be solved in coastal wetland ecosystem and rare species conservation. It was found that low and sparse mangrove forests are vulnerable to the invasion of *S. alterniflora* because it affects their spread. On the other hand, large and lush mangrove forests can shade the growth of *S. alterniflora*, eventually depriving *S. alterniflora* of a suitable environment for survival [113]. At present, various methods have been explored to control *S. alterniflora*, including physical, chemical, and biological methods [114,115,116,117]. According to our results, biological control can be carried out in dense mangrove forest areas in Guangdong, Guangxi, and Hainan. This involves the use of a “*Sonneratia apetala* shade” plantation to control the growth and spread of *S. alterniflora*. In areas where *S. alterniflora* invades mangrove forests, fast-growing *S. apetala* suppresses the growth of *S. alterniflora* through shading and chemosensory effects, while promoting the growth of native mangrove plants to restore the mangrove forest community [118]. In Zhejiang and Fujian, where mangrove forests are sparse and *S. alterniflora* is dense, the growth of *S. alterniflora* can be controlled by physical or chemical methods, including artificial removal, mulching for shade, mowing, fire, flooding, and chemical herbicides [119,120,121,122].

## 4. Materials and Methods

### 4.1. Occurrence Data

**Figure 6:**
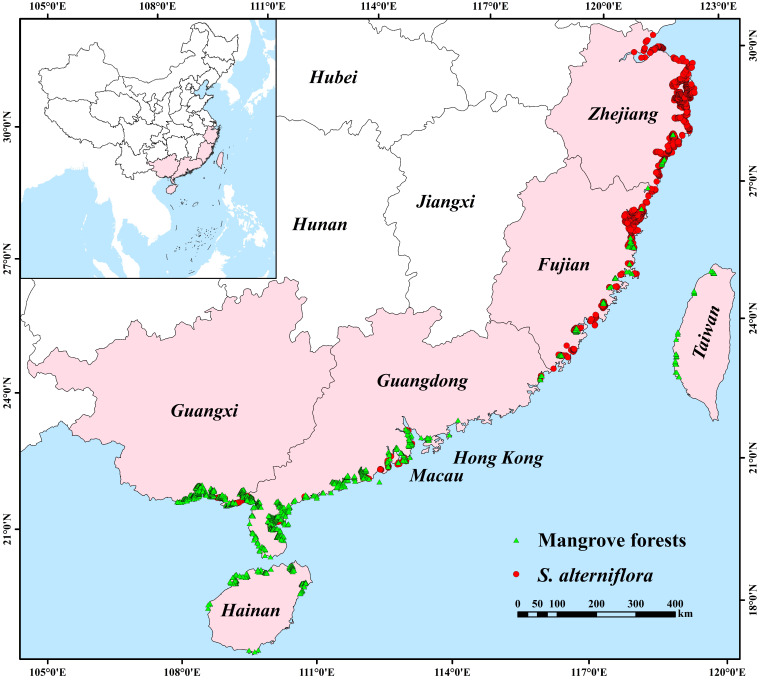
Study area and species occurrence record distribution. The study area mainly includes the provinces of Zhejiang, Fujian, Guangdong, Guangxi, Hainan, Taiwan, Hong Kong, and Macau in China. The green triangles represent the location of mangrove forests. The red dots represent the location of the invasive *S. alterniflora*.

In this study, current mangrove forest distribution areas were selected. The study areas in China include Guangdong, Guangxi, Hainan, Fujian, Hong Kong, Macau, Taiwan, and Zhejiang provinces [87]. Mangrove forest occurrence records were obtained from the spatial distribution dataset of Chinese mangrove forests at 30 m resolution in 2015 (National Earth System Science Data Center (NESSDC), http://www.geodata.cn, accessed on 8 July 2022). Occurrence records of *S. alterniflora* were obtained from the spatial distribution dataset of Chinese *S. alterniflora* at 30 m resolution in 2015 (NESSDC, http://www.geodata.cn, accessed on 22 July 2022). The spatial distribution datasets of mangrove forests and *S. alterniflora* were resampled into the occurrence records with a sampling accuracy of 1 km using the fishnet method in ArcGIS. Then, the collected occurrence records were filtered to eliminate the duplicate records. Finally, 1358 mangrove forest records and 1314 *S. alterniflora* records were obtained for modeling (Figure 6).

### 4.2. Environment Data

We selected three categories of environmental factors, including 20 variables in bioclimate, sea–land topography, and marine environment (Table 2). CHELSA (Climatologies at high resolution for the earth’s land surface areas, https://chelsa-climate.org/, accessed on 24 July 2022) was selected for the bioclimate data, and the elimination of autocorrelation between predictors helped to avoid prediction errors caused by collinearity in bioclimatic variables. Pearson correlation coefficients of 19 bioclimate variables were calculated (Figure S1); only meaningful variables with |*r*| < 0.7 were retained [43], and 9 bioclimate variables were selected to participate in the modeling. The sea–land topography was obtained from the National Marine Data Center (NMDC, http://mds.nmdis.org.cn/, accessed on 28 July 2022). Elevation (Ele), slope (Slop), and aspect (Aspe) were extracted from sea–land topography by ArcGIS. The photosynthetically available radiation (PAR) and chlorophyll concentration (CHL) represent the marine ecological environment and coastal water quality changes. CHL is one of the key indicators of marine primary productivity; it can visually reflect the degree of seawater eutrophication [123,124,125]. The PAR, CHL, and sea surface temperature were obtained from NASA MODIS-Aqua Level-3 (NASA Goddard Space Flight Center, Ocean Ecology Laboratory, Ocean Biology Processing Group (OBPG), http://oceancolor.gsfc.nasa.gov, accessed on 26 July 2022). Sea surface temperature included annual mean sea surface temperature (SST1), sea surface temperature of warmest quarter (SST2), sea surface temperature of wettest quarter (SST3), sea surface temperature of coldest quarter (SST4), and sea surface temperature of driest quarter (SST5). The sea surface salinity (SSS) was obtained from the NMDC (http://mds.nmdis.org.cn/, accessed on 28 July 2022) (Table 2). Saga GIS was used to interpolate the environmental variables and make the range in land and marine variables consistent. All the environmental variables were resampled to a resolution of 30″ (about 1 km) for model prediction and analysis.

### 4.3. Model Construction and Evaluation

This study was completed using the Biomod2 package. We used the ANN, CTA, FDA, GAM, GBM, GLM, MARS, RF, and SRE models in the Biomod2 package on the R platform. To improve the fitting accuracy of the model, three sets of pseudo-absent points were randomly generated. After several experiments, we finally determined that the model had the highest accuracy when generating 5000 pseudo-random points per set. We entered species presence points, pseudo-absence points, and environmental data in the R software environment. Seventy-five percent of the sample data were randomly selected as the training set to build the model, and the remaining twenty-five percent were used for model validation. Each of the nine single models was called and run ten times to reduce uncertainty.

In this study, we evaluated the model using the relative operating characteristic (ROC) curve with the statistics of the AUC value (the area under the ROC curve) and the true skill statistic (TSS). ROC is plotted with 1-specificity (the proportion of species non-occurrence areas correctly predicted) as the horizontal coordinate and sensitivity (the proportion of species occurrence records correctly predicted) as the vertical coordinate [52]. The size of the area under the ROC curve is used to evaluate the ability of the model. A larger value indicates a more accurate prediction, which is one of the indicators for the evaluation of many models [126,127]. TSS (=sensitivity + (specificity-1)) is an improved assay for Kappa that retains the advantages of Kappa [128]. This statistic is one of the default evaluation metrics of the Biomod2 package. In general, when the TSS is greater than 0.8, the AUC is greater than 0.9; this indicates very high accuracy of the model fit [56].

The evaluation results of the single-unit models were obtained, and RF, GBM, and GLM were selected for modeling according to their TSS values which were all higher than other single models. The selected models were run again to obtain 90 single models (3 selected models × 10 repetitions × 3 sets of random datasets). Then, we constructed the EM. All models with TSS values greater than or equal to 0.9 were selected. The weight of the individual model was calculated according to Equation (1), the EM was constructed, and the habitat suitability index [55] of the species was derived using Equation (2).

(1) $$W_{i}=\frac{a_{i}}{\sum_{i=1}^{n}a_{i}}.$$

*W_i_* represents the weight of a single model, *a_i_* represents the TSS value of the *i*th single model, and *n* presents the number of models selected.

(2) $$HSI_{i}=\sum_{j=1}^{n}W_{i}\times P_{ij}.$$

*HSI_i_* represents the habitat suitability index value of each pixel of model *I*; *W_i_* represents the weight of the model *I*; and *P_ij_* represents the *j* pixel value of model *i*.

The habitat suitability index (0–1) output from the model reflects the probability of species presence [9]. According to the statistical principle of “likelihood” in the presence probability analysis [61], we classified the suitability results into four categories [52,55]: unsuitable (*HSI* < 0.3), low suitability (0.3 ≤ *HSI* < 0.5), moderately suitable (0.5 ≤ *HSI* < 0.7), and highly suitable (*HSI* ≥ 0.7).

### 4.4. Risk Assessment of S. alterniflora Invading Mangrove Forests

In order to assess the areas where the distribution between mangrove forests and *S. alterniflora* may shift, that is, the invasive risk of *S. alterniflora* within the mangrove forest distribution area, we used the invasive risk index (*IRI*) (Equation (3)) combined with SDMs simulation results of mangrove forests and *S. alterniflora*. We subtracted the habitat suitability value of the native species (i.e., mangrove forests) from the invasive habitat suitability value of the invasive species (i.e., *S. alterniflora*) to obtain the value of *IRI* of *S. alterniflora* in the study area.

(3) $$IRI=HSI_{s}-HSI_{m}.$$

*IRI* represents the invasive risk index with thresholds ranging from −1 to 1; *HSI_s_* represents the result of the SDM simulation of *S. alterniflora*; and *HSI_m_* represents the result of the SDM simulation of mangrove forests. A positive value of *IRI* indicates that the region is more suitable for the growth of *S. alterniflora*. The invasive risk of *S. alterniflora* in this region is high, which may affect the growth and renewal of local mangrove forests. A negative value suggests that the habitat suitability of mangrove forests in this region is higher, and the invasive risk of *S. alterniflora* is lower.

## 5. Conclusions

*S. alterniflora* poses a serious threat to native coastal ecosystems due to its high environmental adaptability and rapid population spread. The strong vitality of *S. alterniflora* has led to the disappearance of large areas of mangrove forests because it crowds out mangrove forest growing space. We used EM to simulate the geographic distribution of mangrove forests and *S. alterniflora* in southeastern China and deduced the environmental threshold for their growth. On this basis, we assessed the invasion risk of *S. alterniflora* to mangrove forests along the southeast coast. According to our analysis, mangrove forests are mainly concentrated in southern Guangxi, Guangdong, and *S. alterniflora* is mainly distributed in Zhejiang and Fujian. We should strengthen the control of *S. alterniflora* and the introduction and cultivation of mangrove forests in the coastal areas of Zhejiang, Fujian, Beibu Gulf, and Zhuhai, Guangdong. Mangrove forest restoration projects should be increased in the coastal areas of Guangdong and southern Guangxi. Our study provides a clear understanding of the geographic distribution of mangroves and *S. alterniflora* and the high-risk areas where *S. alterniflora* is more likely to invade mangrove forests. We believe that this study can provide a basis for mangrove forest conservation and *S. alterniflora* control along the southeast coast of China.

## Figures and Tables

**Table 1:** Contribution rate and cumulative contribution rate of each environmental variable based on the ensemble model.

Mangrove Forests				*S. alterniflora*			
Variables	Contribution Rate (%)	Cumulative Contribution Rate (%)	Best Suitable Range (Unit)	Variables	Contribution Rate (%)	Cumulative Contribution Rate (%)	Best Suitable Range (Unit)
Bio1	25.07	25.07	18.7–25.7 °C	CHL	22.34	22.34	3.04–13.85 µg/L
Ele	17.86	42.93	−140–16 m	Bio16	21.27	43.61	438–1226 mm
CHL	17.84	60.77	1.17–13.14 µg/L	Ele	8.29	51.90	−149–165 m
Bio16	7.63	68.40	438–1642.76 mm	Bio19	7.05	58.95	69–332 mm
Bio12	7.30	75.70	949–2669 mm	Bio3	6.34	65.29	116–276
SST2	5.14	80.84	25.41–38.4 °C	Bio12	6.08	71.37	932–2491.4 mm
Bio19	3.19	84.03	23.6–566 mm	Bio2	6.06	77.43	25–70
PAR	3.18	87.21	28.13–39.56 E/m^2^day	PAR	5.92	83.35	26.25–35.48 E/m^2^day
Bio15	2.63	89.84	20–100 mm	Bio18	3.68	87.03	302–1137 mm
Bio2	2.54	92.38	20–62	Bio15	2.61	89.64	32–87 mm
Bio18	1.83	94.21	302–1586.94 mm	SST1	2.31	91.95	8.8–35.4 °C
SSS	1.65	95.86	30.9–33.9%	Bio5	1.91	93.86	28.4–33.3
Bio5	1.31	97.17	29.7–32.5	Bio1	1.75	95.61	16.9–23.8 °C
Bio3	1.08	98.25	150–297	SST2	1.16	96.77	22.1–37.3 °C
SST4	0.89	99.14	13.29–32.03 °C	SSS	1.04	97.81	21.4–33.1%
SST3	0.31	99.45	19.82–36.87 °C	Slop	0.75	98.56	0–90°
SST1	0.29	99.74	16.6–36.5 °C	SST3	0.75	99.31	15.9–36.1 °C
SST5	0.14	99.88	22.39–34.9 °C	SST4	0.56	99.87	8.8–31.1 °C
Slop	0.10	99.98	0–90°	SST5	0.10	99.97	15.88–34.42 °C
Aspe	0.02	100.00	−1–359.82°	Aspe	0.03	100.00	−1–359.82°

**Table 2:** Environmental variables used to simulate predictions of mangrove forests and *S. alterniflora*.

Factors	Variables	Description	Unit	Data Sources
BioClimate	Bio1	Annual mean temperature	°C	CHELSA (https://chelsa-climate.org/ (accessed on 12 March 2023))
	Bio2	Mean diurnal range	°C	
	Bio3	Isothermality	-	
	Bio5	Max temperature of warmest month	-	
	Bio12	Annual precipitation	mm	
	Bio15	Precipitation seasonality	mm	
	Bio16	Precipitation of wettest quarter	mm	
	Bio18	Precipitation of warmest	mm	
	Bio19	Precipitation of coldest quarter	mm	
Sea–land topography	Ele	Elevation	m	National Marine Data Center (http://mds.nmdis.org.cn/ (accessed on 12 March 2023)), Slop and Aspe are extracted from the sea–land topography by ArcGIS.
	Slop	Slope	°	
	Aspe	Aspect	°	
Marine environment	CHL	Chlorophyll concentration	µg/L	NASA MODIS-Aqua Level-3 (http://oceancolor.gsfc.nasa.gov (accessed on 12 March 2023))
	PAR	Photosynthetically available radiation	E/m^2^day	
	SSS	Annual mean sea surface salinity	%	National Marine Data Center (http://mds.nmdis.org.cn/ (accessed on 12 March 2023))
	SST1	Annual mean sea surface temperature	°C	NASA MODIS-Aqua Level-3 (http://oceancolor.gsfc.nasa.gov (accessed on 12 March 2023))
	SST2	Sea surface temperature of warmest quarter	°C	
	SST3	Sea surface temperature of wettest quarter	°C	
	SST4	Sea surface temperature of coldest quarter	°C	
	SST5	Sea surface temperature of driest quarter	°C	

## Data Availability

The data presented in this study are openly available in Mendeley Data at doi: 10.17632/759yn23jb9.1.

## Supplementary Materials

The following supporting information can be downloaded at: https://www.mdpi.com/2223-7747/12/10/1923/s1, Figure S1: Correlation analysis of 19 bioclimate variables. The color and size of the circles represent the magnitude of the correlation between the variables. The upper numbers and the lower circles correspond variables with one another; Figure S2: Variables with a cumulative contribution greater than 90% affecting the growth of mangrove forests and *S. alterniflora*. Blue represents the contribution rate of mangrove forests and pink is the contribution rate of *S. alterniflora*.
