# Supplementary material for: Invasive Trends of Spartina alterniflora in the Southeastern Coast of China and Potential Distributional Impacts on Mangrove Forests
Source: Plants (Basel). 2023 May 9;12(10):1923. doi: 10.3390/plants12101923 (PMC10222674; doi:10.3390/plants12101923)
Supplement: Supplementary file 1 [file plants-12-01923-s001.zip › plants-2310545-supplementary.pdf]

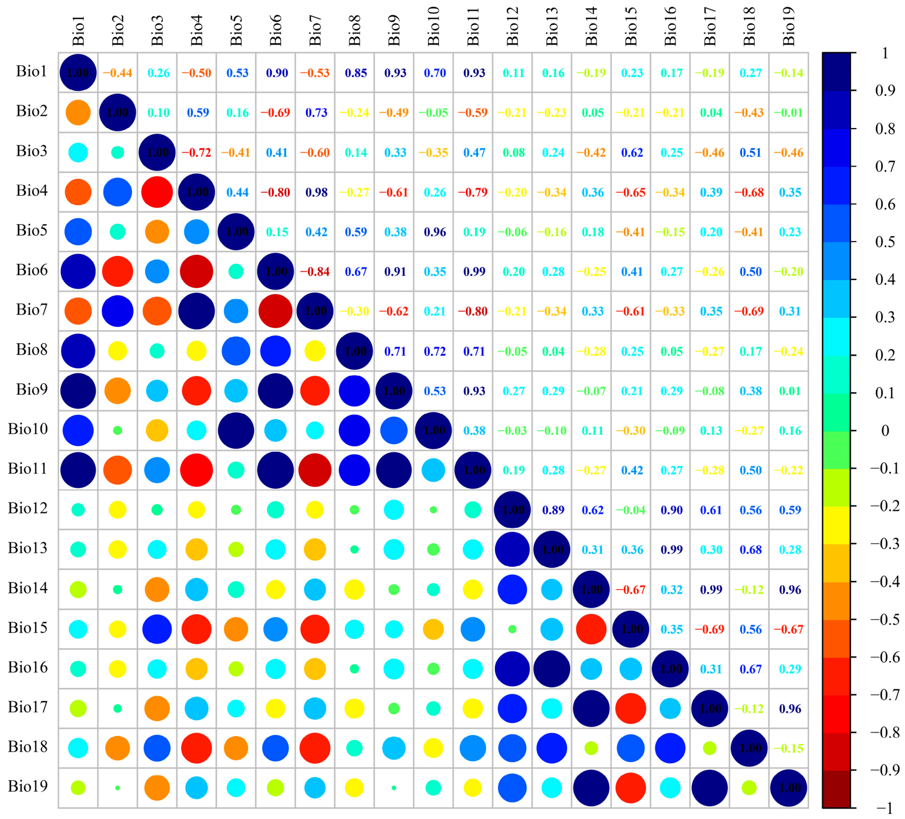

Figure S1. Correlation analysis of 19 bioclimate variables. The color and size of the circles represent the magnitude of the correlation between the variables. The upper numbers and the lower circles correspond variables one to another.

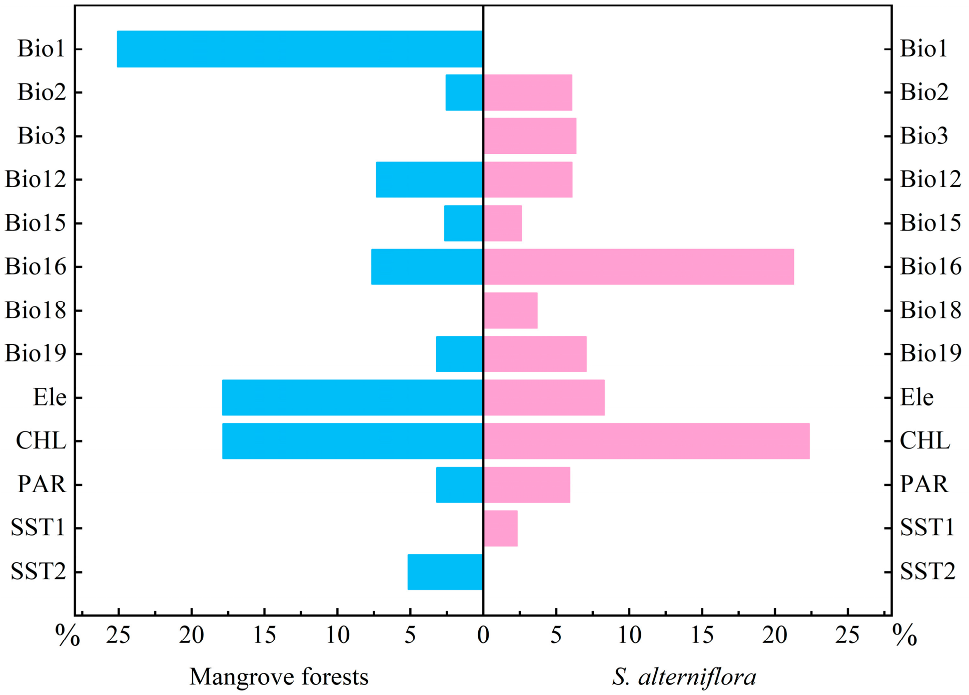

Figure S2. Variables with a cumulative contribution greater than 90% affecting the growth of mangrove forests and *S. alterniflora*. Blue represents the contribution rate of mangrove forests and pink is the contribution rate of *S. alterniflora*.
